# Identification and genetic analysis of *qCL1.2*, a novel allele of the “green revolution” gene *SD1* from wild rice (*Oryza rufipogon*) that enhances plant height

**DOI:** 10.1186/s12863-020-00868-w

**Published:** 2020-06-11

**Authors:** Lizhen Zhang, Jingfen Huang, Yanyan Wang, Rui Xu, Ziyi Yang, Zhigang Zhao, Shijia Liu, Yunlu Tian, Xiaoming Zheng, Fei Li, Junrui Wang, Yue Song, Jiaqi Li, Yongxia Cui, Li-Fang Zhang, Yunlian Cheng, Jinhao Lan, Weihua Qiao, Qingwen Yang

**Affiliations:** 1 Qingdao Agricultural University, Qingdao, 266109 China; 2 Institute of Crop Science, Chinese Academy of Agricultural Sciences, Beijing, 100081 China; 3 Shanxi Agricultural University, Shanxi province, Taigu, China; 4 Nanjing Agricultural University, Nanjing, 571100 China

**Keywords:** Wild rice, Chromosome segment substitution line, *SD1*, Plant height, Rice domestication

## Abstract

**Background:**

The exploitation of novel alleles from wild rice that were lost during rice cultivation could be very important for rice breeding and evolutionary studies. Plant height (PH) was a target of artificial selection during rice domestication and is still a target of modern breeding. The “green revolution” gene semi-dwarf 1 (*SD1*) were well documented and used in the past decades, allele from wild rice could provide new insights into the functions and evolution of this gene.

**Results:**

We identified a PH-related quantitative trait locus, *qCL1.2*,from wild riceusing a set of chromosome segment substitution lines. *qCL1.2*encodesa novel allele of *SD1* gene. The wild allele of *SD1* is a dominant locus that can significantly promote rice internode length by regulating the expression levels of genes involved in gibberellin biosynthesis and signal transduction. Nucleotide diversity and haplotype network analyses of the *SD1* gene were performed using 2822 rice landraces. Two previously reported functional nucleotide polymorphisms clearly differentiated *japonica* and *indica* rice; however, they were not associated with PH selection. Other new functional nucleotide polymorphisms in the coding, but not promoter, regions were involved in PH selection during rice domestication. Our study increasesunderstanding of the rice *SD1* gene and provides additional evidence of this gene’s selection during rice domestication.

**Conclusions:**

Our findings provide evidence that*SD1* gene from wild rice enhances plant height and new functional nucleotide polymorphisms of this gene were artificially selected during cultivated rice differentiation.

## Background

Asian rice (*Oryzasativa*L.) is a cultivated, inbred species that provides 35–60%ofdietarycaloriesto ~ 50% of the world’s population [1].Plant-architectureis crucial to the high yield of rice, and the ideal plant-architectureis essential for a high rice yield [2, 3]. Plant height (PH) is a main factor affecting rice plant-architecture, and an ideal PH is necessary for a high crop yield. PH is mostly determined by cell division, cell differentiation and cell expansion in the stem. Plant hormones, such as gibberellin (GA), play important roles in PH regulation [4]. In the 1960s, a mutation in the Taiwanese *indica* landrace ‘Dee Gee Woo Gen’ [5] led to a semi-dwarf variety of rice, known as IR8, which made an outstanding contribution to world food security, known as the rice “green revolution” [6, 7]. The short PH of IR8 results from a mutation in the plant’s semi-dwarf 1 (*sd1*) gene, which islocated on the long arm of chromosome (Chr.) 1 and encodes an oxidase enzyme involved in GA biosynthesis [8]. A recessive allele, *sd1*, caused by a 383-bp deletion in *SD1*, is primarily responsible for the reduction in PH observed in most semi-dwarfs [5, 8, 9]. At present, at least five different alleles, including the wild-type allele, *sd1-d* in ‘Dee Gee Woo Gen,‘*sd1-r* in ‘Reimei,’ *sd1-c* in ‘Calrose76’ and *sd1-j* in ‘Jikkoku’ have been discovered [5, 9]. Mutants of these alleles lead to different degrees of dwarfing through changes in PH. However, the origin of the rice *sd1* allele and the role of *sd1* in rice domestication are still unclear.

Common wild rice (*Oryza rufipogon* Griff.), which has an AA genome similar to that of cultivated rice, is considered the ancestor of cultivated rice [10–12]. Wild rice has a greater genetic diversity than cultivated rice because genetic diversity was profoundly reduced during rice domestication [13]. Many novel alleles of genes controlling important agronomic traits in rice have been found in wild rice and its relatives, and they have provided an increased understanding of gene functions and the domestication process [14]. The development of chromosome segment substitution lines (CSSLs) through interspecific hybridization is a powerful platform for QTL mapping and gene cloning and produces useful genetic resource for genome research [15]. In our previous study, a set of CSSLs was constructed with wild rice as the donor parent and the *indica* cultivar 9311 as the recurrent parent. Many quantitative trait loci (QTLs) correlated with important agronomic traits have been identified using the CSSL platform [16–18]. In this study, we fine mapped a novel allele of the “green revolution” gene *sd1* using a CSSL population. The genetic analysis revealed that this allele from wild rice is a dominant locus that can significantly increase rice culm length. A previous report [19] suggested that *sd1* was subjected to artificial selection during rice evolution, and two single nucleotide polymorphisms (SNPs) of *sd1* can clearly differentiate the *japonica* landraces and wild rice. Nucleotide diversity and haplotype network analyses of the *sd1* geneconfirmed this hypothesis. However, these two SNPs were not associated with the PH phenotype. We found nine other functional nucleotide polymorphisms (FNPs) that were used in rice domestication owing to their influence on PH. Our study presents new evidence for artificial PH selection during rice domestication and differentiation, and the novel *SD1* allele and the FNPs provide an increased understanding of rice PH-targeted breeding.

## Results

### *q*CL1.2 detection using a CSSL population

In previously study, a set of CSSLs was constructed in our laboratory [10] . The donor wild rice parental plant has a procumbent phenotype. To identify genes controlling PH during rice domestication, we conducted a QTL analysis for PH using this CSSLs population. The PHs of the CSSLs were investigated under five environmental conditions (Table 1). The PH phenotype substantially differed within the CSSL population (Fig. S1). Genotyping was performed using 157 molecular markers, including 97 SSR markers and 60 InDel markers. The linkage map of SSR/InDel markers was shown in Fig. S2a. In total, 11 QTLs correlated with PH were identified under the five environmental conditions (Table 2). One QTL, located near InDel 1–16 on Chr. 1 was detected in four environments, had the highest LOD value (45.01 in E3) and explained 48% of the PH variance (Table 2), indicating that this QTLis likely a main effect QTL. This QTL was named as *qCL1.2.* One CSSL, CSSL28, which had the greatest PH in the CSSL population and harbored*qCL1.2*, was selected for further study. The CSSL28 genotype is shown in Fig. S2a. Only two substituted segments from wild rice were detected using SSR/InDel markers in the whole CSSL28 genome (Fig. S2a); therefore, CSSL28 was considered a near isogenic line (NIL) of *qCL1.2*.

**Table 1:** The locations of rice crops used in this experiment

Environment	Crop location	Cropping season
E1	Shunyi, BeijingN40.20°, E115.51°	Apr-Oct. 2017
E2	Nanjing, Jingsu Province,N32.03°, E118.47°	May-Oct. 2017
E3	Sanya, Hannan Province,N18.15°, E109.31°	Dec.2017 -May 2018
E4	Shunyi, Beijing, N40.20°, E115.51°	April-Oct. 2018
E5	Nanjing, Jingsu Province, N32.03°, E118.47°	April-Oct. 2018

**Table 2:** QTLs correlated with plant height in five environments identified using SSR/InDel genotypes detected in a CSSL population

Marker	Chr.	Environment	LOD	PVE(%)	Add
InDel1–16	1	E3	45.01	48.01	40.76
		E1	40.12	44.02	48.66
		E2	31.86	40.94	41.38
		E5	3.263	4.830	12.95
RM125	7	E3	14.85	11.04	22.76
		E5	13.67	10.97	28.28
RM427	7	E3	8.480	5.869	−20.83
		E5	8.105	6.106	−26.49
RM5427	6	E5	4.071	8.665	0.6553
InDel4–3	4	E1	6.979	6.726	−22.43
InDel6–4	6	E2	6.886	10.60	−62.14
InDel1–12	1	E2	5.409	8.197	−38.72
RM190	6	E1	4.635	4.353	−21.99
RM128	1	E4	4.438	9.150	32.54
RM273	4	E1	4.265	3.988	24.25
RM533	7	E2	2.774	4.084	17.41

PVE, the percentage of phenotypic variation explained; Add, the additive effect of the QTL

### Phenotypic characteristics of parental lines CSSL28 and 9311 and their F_1_ generation

**Fig. 1:**
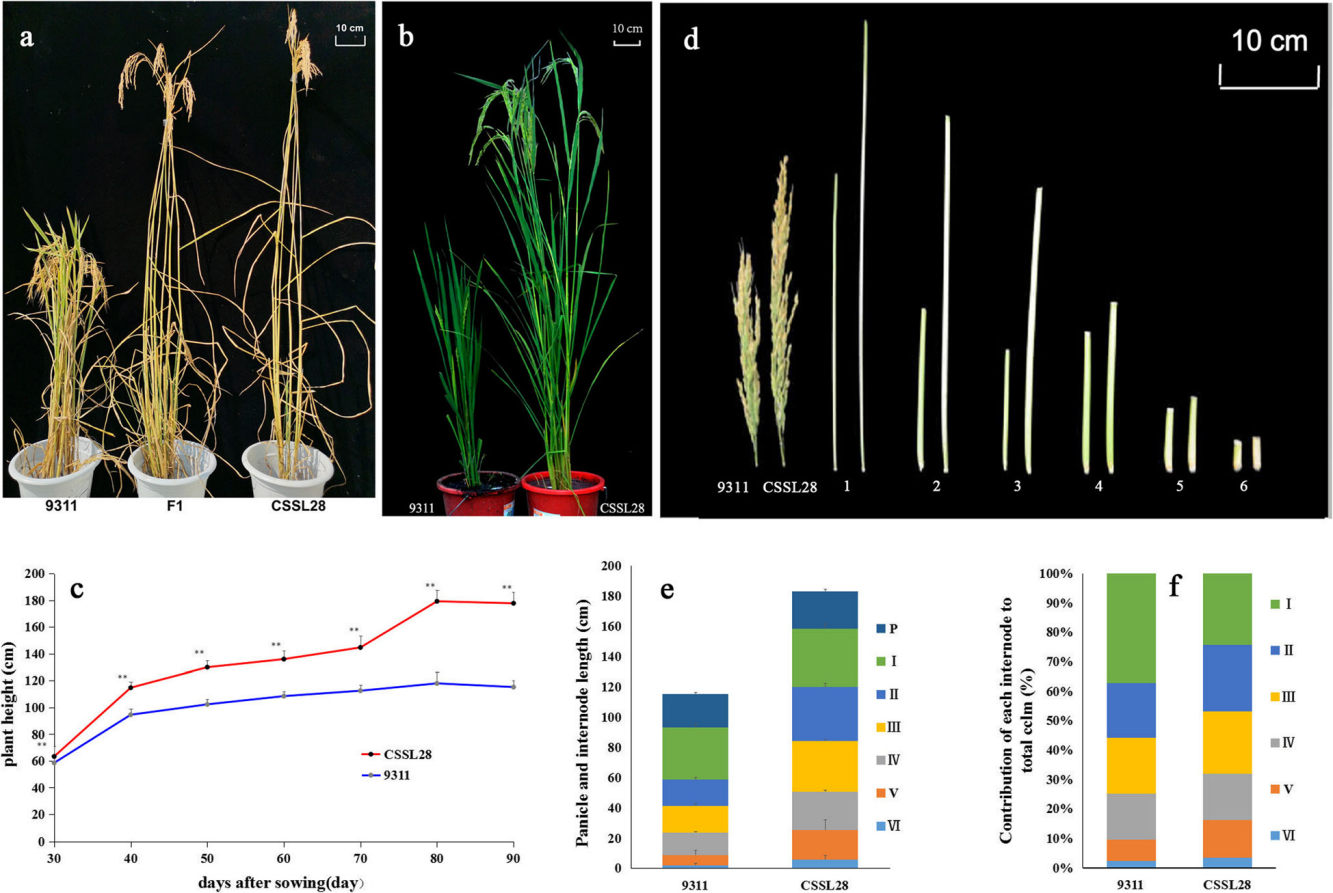
Gross morphology of CSSL28 and 9311 rice lines. **a** and **b** Plant phenotypes of CSSL28, 9311 and individuals from the CSSL28/9311 F_1_ generation. **c** Dynamic comparison of plant heights between CSSL28 and 9311 at different growth stages; all data are provided as means ± SDs (*n* = 20). **d** The appearances of the panicles and internodes of CSSL28 and 9311; 1–6 indicate internodes from head to base. **e** Comparison of the lengths of the panicles and internodes between CSSL28 and 9311; data are averages of the lengths of the panicles and internodes of the main culms (*n* = 50). **f** Schematic representation of internode elongation patterns of CSSL28 and 9311. **significant at *P* < 0.01

CSSL28 showed a significantly greater PH than the recurrent parent 9311. The PH of CSSL28 was 180.3 cm, while that of 9311 was 116.8 cm (in E5). The F_1_ was generated from a cross with CSSL28 as the female parent and 9311 as the male parent. The resulting F_1_ individuals were as tall as CSSL28 (Fig. 1a, b). A difference in PH between CSSL28 and 9311 was clear evident at the seedling stage (Fig. S2b), The difference was significant from30 d after sowing. The difference in PH between CSSL28 and 9311 was extremely significant at the heading stage, reaching ~ 63 cm on average (Fig. 1c). The lengths of panicle and internodes of 9311 and CSSL28 were also measured (Fig. 1d, e). The basal three internodes of CSSL28 were similar in length to those of 9311. However, the upper three internodes and panicles of CSSL28 were longer than those of 9311. The second and third internodes of CSSL28 were longer than those of 9311 by ~ 18.4 and ~ 17.7 cm, respectively. The total lengths of second and third internodes in CSSL28 contributed approximately 43.8% to the total culm, as compared with 37.6% in 9311 (Fig. 1f). The increase in CSSL28 PH was mainly caused by elongated upper second and third internodes.

**Fig. 2:**
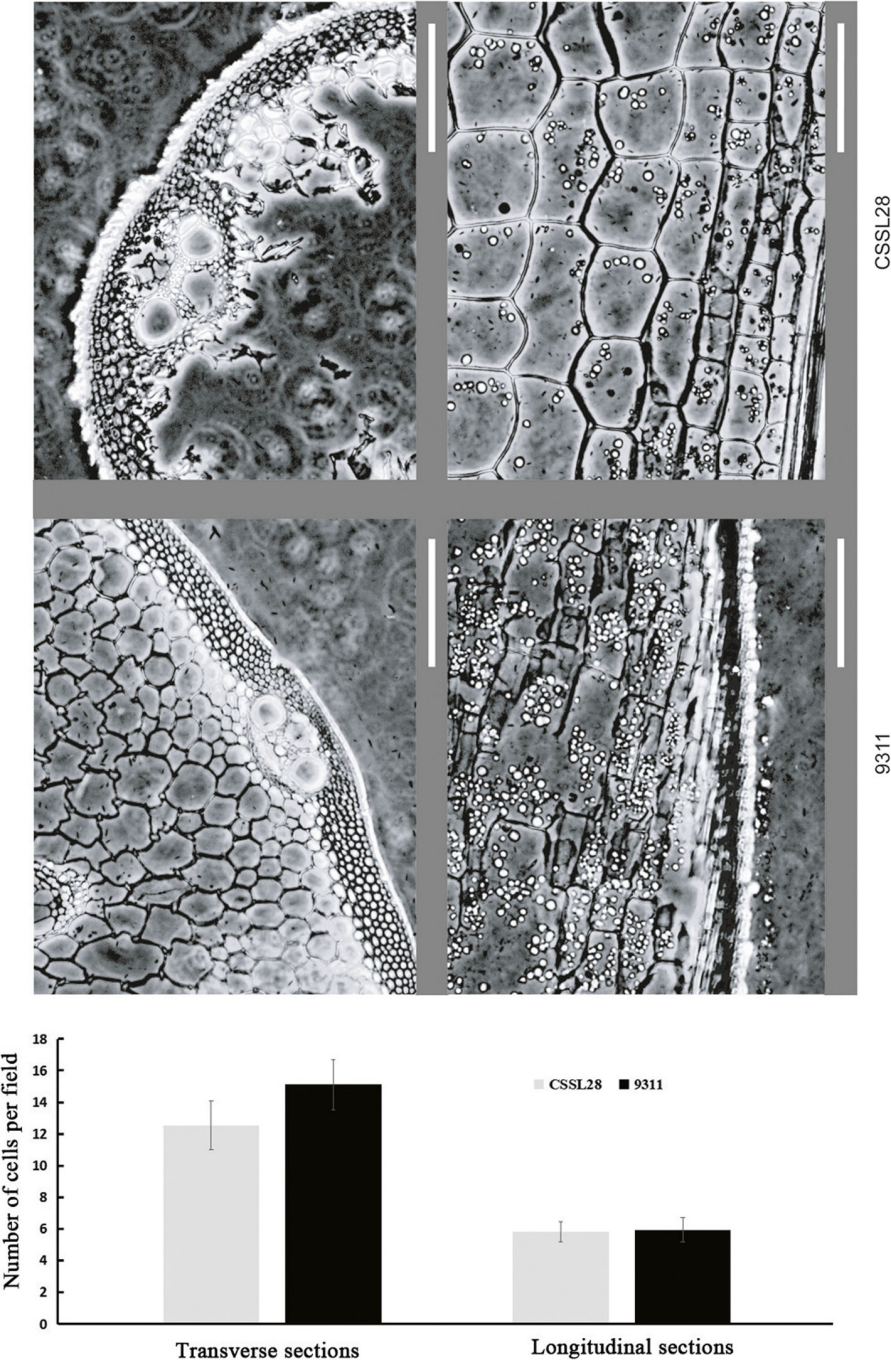
Morphological characterization of the stems of CSSL28 and 9311 rice plants. Transverse and longitudinal sections of the third internode from the main culm at the heading stage. The statistical comparisons of the numbers of cells per field between CSSL28 and 9311 are shown below

To determine the cause of the differences in PH, histological observations of transverse and longitudinal sections of the internodes of CSSL28 and 9311 were recorded (Fig. 2). The transverse sections of the third internodes from the main culms indicated that the CSSL28 cells, especially the vascular cells, were much bigger than those of 9311. The longitudinal sections of the internodes suggested that there was no significant difference in cell length between CSSL28 and 9311. Similar results were observed for the second, fourth and fifth internodes. However, for the first and basal internodes, no differences between CSSL28 and 9311 were observed in the transverse sections. Because the stems of CSSL28 are much thicker than those of 9311, we deduced that the increased PH of CSSL28 resulted from an enhanced cell number and cell size at the first through fifth internodes, rather than an enhanced cell length.

### Fine mapping of *qCL1.2* and gene prediction

**Fig. 3:**
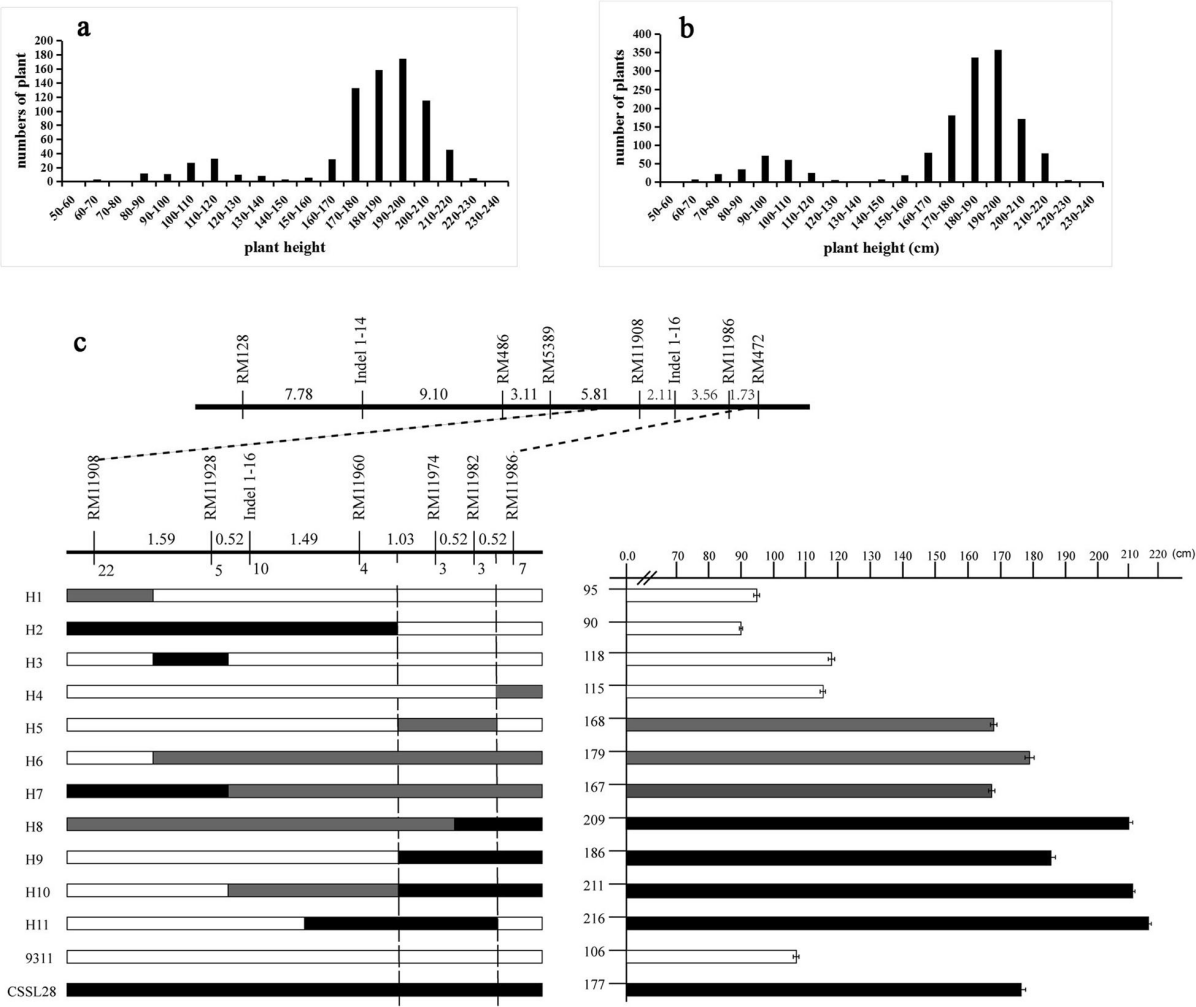
Fine mapping of *qCL1.2* in rice. **a** and **b**Frequency distributions of plant height in segregating populations cultured in Environments 3 (**a**) and 5 (**b**), Environments information were showed in Table 1. **c** The location of *qCL1.2* was narrowed to a 131-kb interval between RM11974 and RM11982. The genetic distance (cM) between two neighbouring markers is indicated above the marker label. The number of *qCL1.2* recombinants obtained is indicated under the marker labels. On the left, the number of individuals of each haplotype are shown; on the right, the average plant heights are shown. *P*-values were calculated using Student’s t-test

The F_2_ population of CSSL28/9311, containing 402 individuals, was constructed for a genetic analysis in the summer of 2017. Thesegregation ratio of PH fit a 3:1 ratio (χ^2^ = 1.76 < χ^2^0.05,1 = 3.84) for single gene inheritance. In2018, two segregating F_3_ populations derived from a single heterozygous plant were used for further genetic analyses. One F_3_ population, containing 1611 individuals, was planted in E3, and another one, containing 928 individuals, was plant in E5. As shown in Fig. 3a, b, the PH showed a bimodal distributionand similar 3:1 segregation ratios were obtained (χ^2^ = 2.20 < χ^2^0.05,1 = 3.84, χ^2^ = 3.31 < χ^2^0.05,1 = 3.84). These results indicated that the difference in PH between CSSL28 and 9311 was controlled by a single QTL, *qCL1.2*.

We located *qCL1.2* between RM128 and RM472 (near InDel 1–16) on Chr. 1. To narrow the site of *qCL1.2* into a smaller region, we selected molecular markerswithin this interval. One InDel and eight SSR markers (InDel1–14、RM486、RM5389、RM11908、RM11986、RM11928、RM11960、RM11974、RM11982) with polymorphisms between CSSL28 and 9311 were seleced. Using ~ 2000 F_3_ segregating individuals, *qCL1.2* was narrowed to a 131-kb interval between RM11974 and RM11982 (Fig. 3c). According to the Rice Genome Annotation Rice Genome Annotation Project Database (http://rice.plantbiology.msu.edu/), this interval may include 13 candidate genes (Table S1), including the “green revolution” gene *sd1* (*LOC_Os01g66100*). By sequencing *LOC_Os01g66100*,wefound that the first and third exons in the coding region produced synonymous and non-synonymous SNP changes, respectively, which altered the tyrosine in CSSL28 to a termination codon in 9311 (Fig. S3). In addition, the promoter region was also altered at 17 sites between CSSL28 and 9311. Functional defects in the *SD1* gene result in serious PH changes. Therefore, we hypothesized that *qCL1.2* is the *SD1* gene and that the extremely high PH of CSSL28 results from thewild rice*SD1* allele.

### Gene expression analysis

**Fig. 4:**
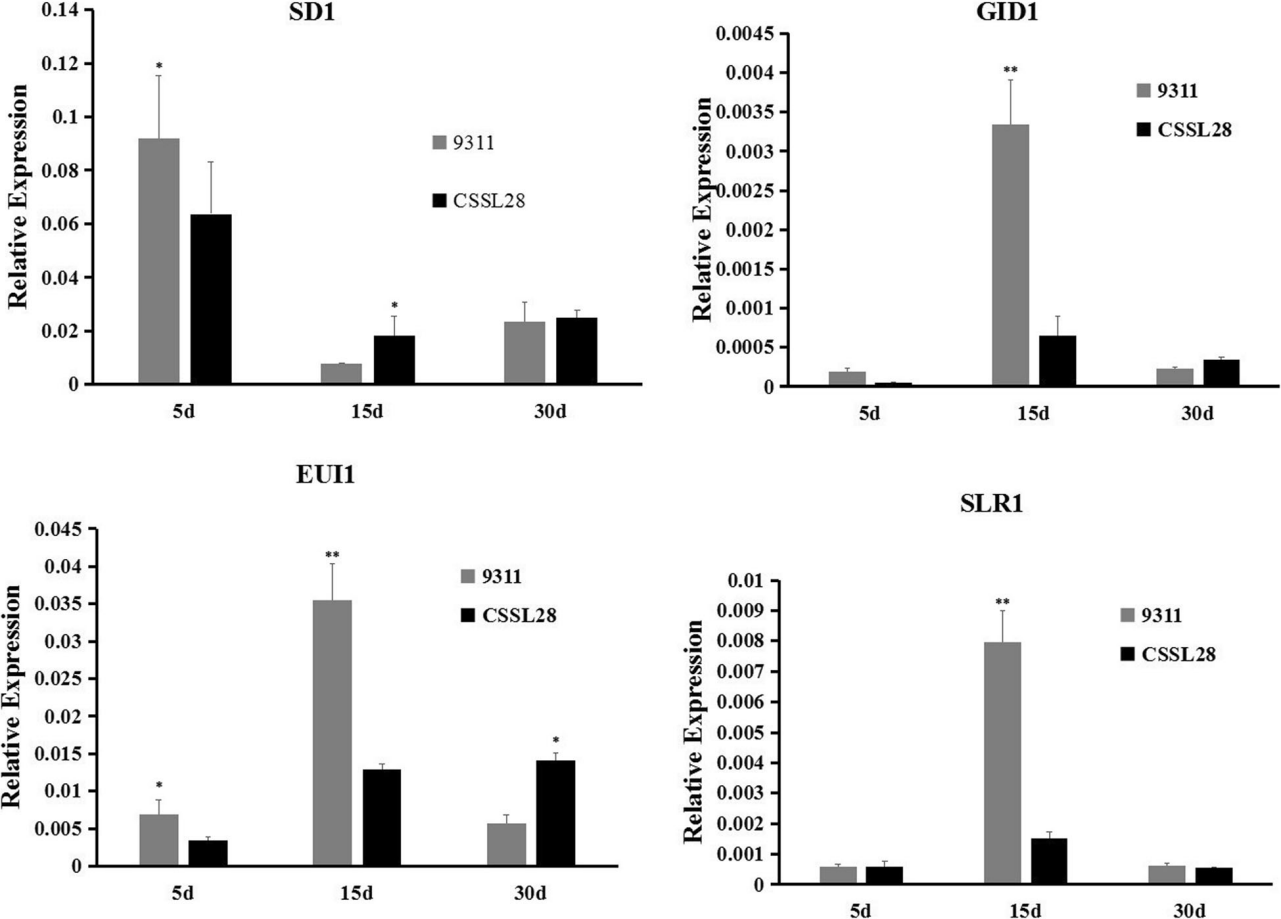
Expression analysis of GA-regulated genes. Total RNA was extracted from CSSL28 and 9311 rice seedlings at 5, 15 and 30 days after germination. **significant at *P* < 0.01

To investigate the expression patterns and regulatory network of the novel allele of the *SD1* gene, total RNA from seedlings of CSSL28 and 9311 at 5, 15 and 30 d after germination were isolated for a real-time PCR analysis. *SD1* and genes involved in GA synthesis (*EUI1*) and GA signaling (*SLR1* and *GID1*) were selected (Fig. 4). For *SD1*, the expression level was high at 5 d after germination in both CSSL28 and 9311, and the expression level in 9311 was higher than that in CSSL28. At 15 and 30 d into the seedling stage, the expression level of *SD1* decreased in both CSSL28 and 9311.

For the*SLR1* gene, which encodes a *DELLA* protein, and the GA receptorgene *GID1*, the expression levels were low at 5 d after germination and significantly increased at the 15th day of the seedling stage. The expression levels of the two genes in 9311 were much higher than those in CSSL28 and then significantly decreased by the 30th day of the seedling stage. The same expression patterns were also found for the *EUI1* gene, which correlates with the internode lengths at the top of rice stems.

### Nucleotide diversity and haplotype network analyses of the *sd1* gene

**Fig. 5:**
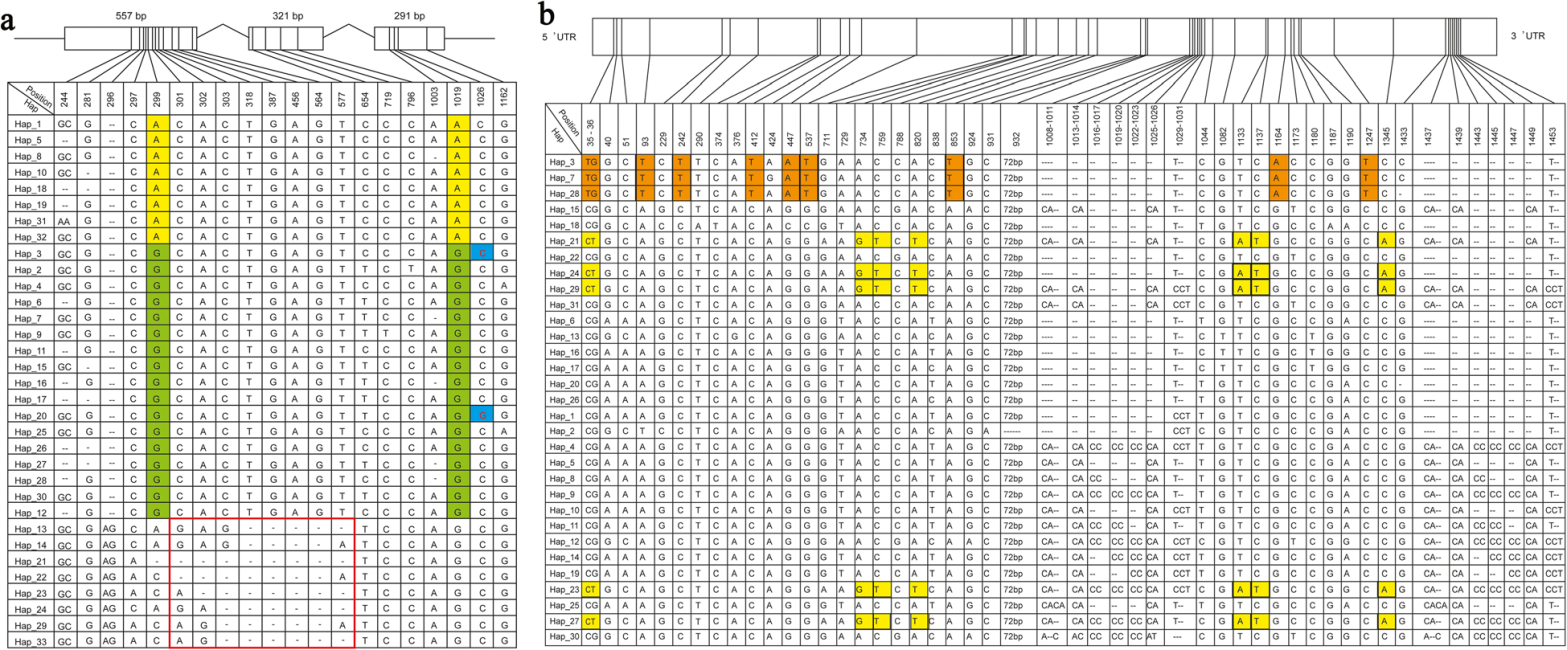
Haplotype analysis of the *SD1* gene in rice. **a** Major haplotypes (haplotypes carried by more than five accessions) of the *SD1* coding region in the whole population based on non-synonymous SNPs data. Different colors at nucleotides 299 and 1019 represent *japonica* and *indica*. H_3 contains *qCL1.2*, the SNPs in blue and red rectangles represent FNPs for plant height. **b** Major haplotypes (haplotypes carried by more than 10 accessions) of the *SD1* promoter region. SNPs in orange differentiate between *japonica* and *indica*, SNPs in yellow are specific to other accessions

**Fig. 6:**
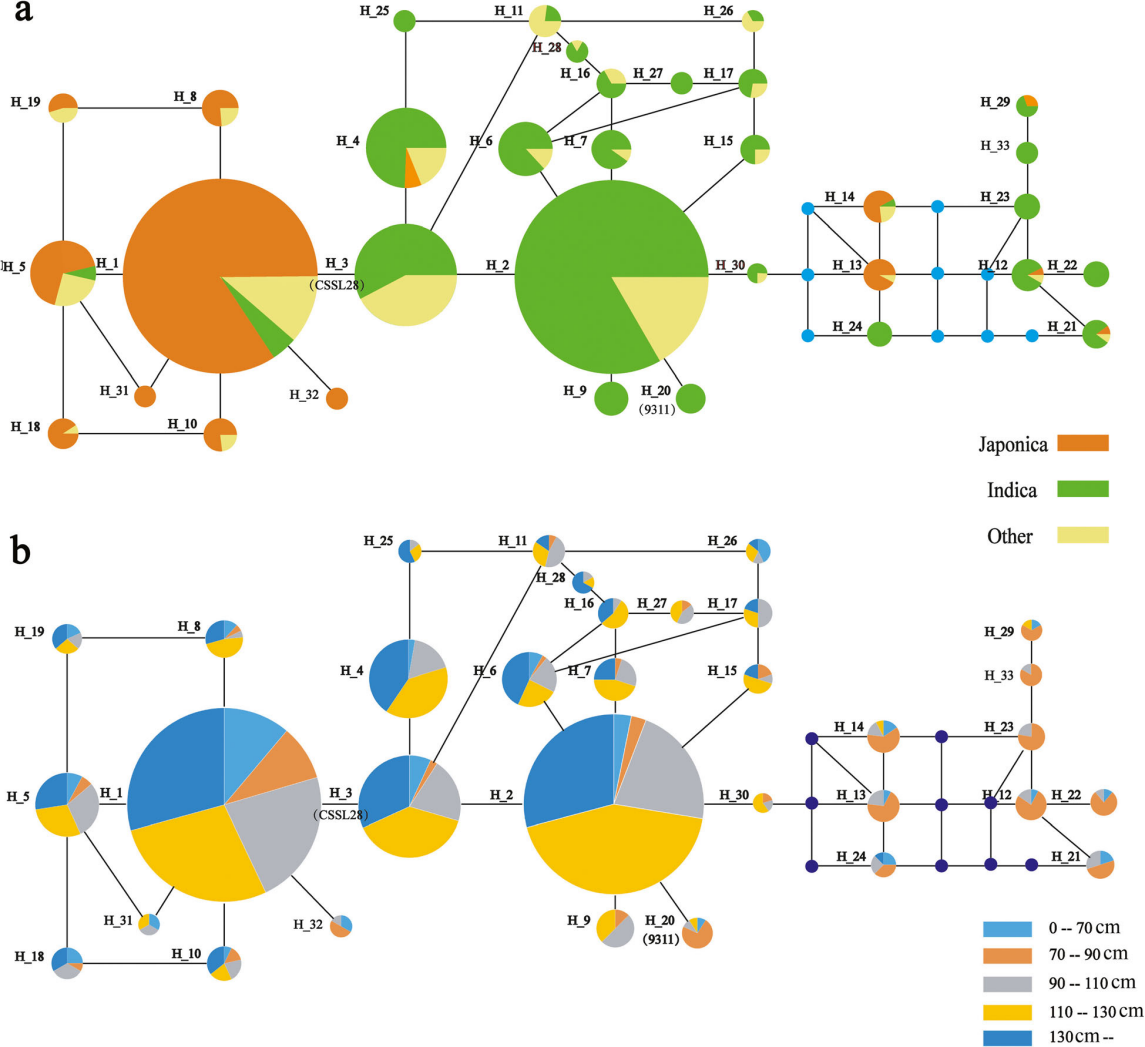
Haplotype and plant height networks based on the CDS region of the *sd1* gene in rice. Circle size is proportional to sample quantity within a given haplotype. Lines between haplotypes represent mutational steps between alleles

Using the rice functional genomics-based breeding database (http://www.rmbreeding.cn/index), the *qCL1.2* (*SD1*) gene coding and promoter region sequences from 2822 rice varieties were aligned. Haplotype and genetic diversity analyses were carried out using the data of 2822 cultivated rice PH phenotypes. Abundant genetic variations were detected at the *LOC_Os01g66100* site in the 2822 cultivated rice accessions (Fig. 5). The *SD1* coding region contained 27 non-synonymous SNP/InDel sites. In total, 33 haplotypes with more than 5 individuals were selected, and a total of 20 variation sites were retained (Fig. 5a). As shown in Fig. 6, a network was constructed using the major haplotypes for the *SD1* coding region. The 33 haplotypes were basically divided into three groups. The left group contained 8 haplotypes and 95.5% of the *japonica* rice samples, and the middle group contained 16 haplotypes and 89.1% of the *indica* rice samples (Fig. 6a*)*. Associations between haplotypes and PH were also analyzed (Fig. 6b). Among the 24 haplotypes in the left and middle groups,97.6% accessions having PH values greater than 130 cm, and 95.5% of the samples having PH values between 110 and 130 cm were in this group. In the right group, most of the PH values were less than 90 cm, and 50.69% of samples having PH values between 70 and 90 cm were in this group.

As shown in Fig. 5a, SNPs at nt 299 and 1019 in the *SD1* coding region differentiated *japonica* and *indica* rice. The amino acids at the two sites were glutamate (E) and glutamine (Q), respectively, in *japonica*, and glycine (G) and arginine (R), respectively, in *indica.* More than 98% of the *indica* accessions carried the SD1-GR allele, as in *qCL1.2*. Most of the *japonica* accessions carried the SD1-EQ allele. However, these two SNPs did not affect PH. Compared with other haplotypes, the InDels in haplotypes H_13, H_14, H_21, H_22, H_23, H_24, H_29 and H_33 resulted in frame-shifts or translational termination, leading to the dwarf plant phenotype. Additionally, in the H_20of 9311, the SNP at nt 1026 led to dwarfed plants.

**Fig. 7:**
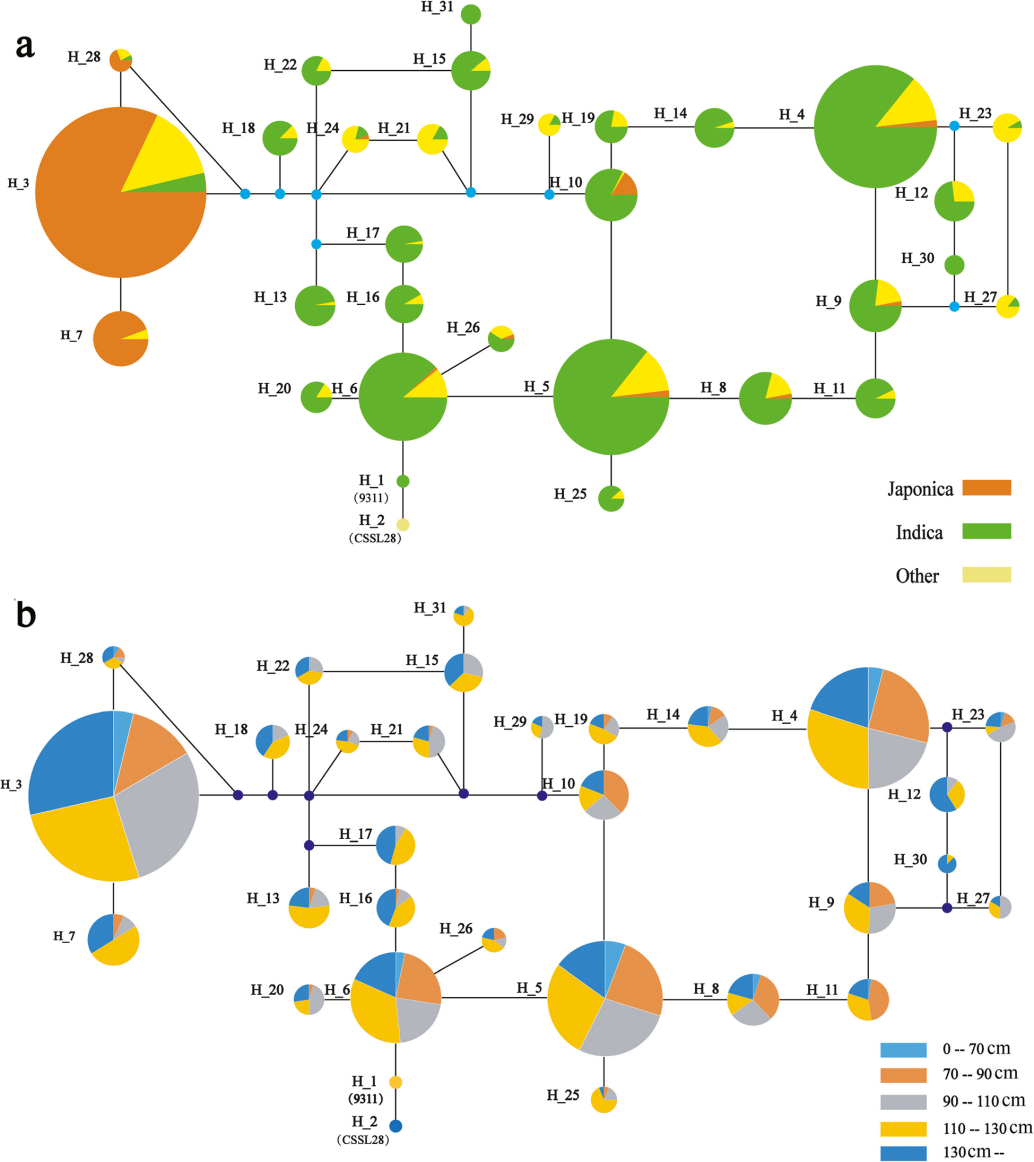
Haplotype and plant height networks based on the promoter region of the *sd1* gene in rice. Circle size is proportional to sample quantity within a given haplotype. Lines between haplotypes represent mutational steps between alleles

A network for the *SD1* promoter sequence was also constructed using the same database. The *SD1* promoter region contained 51 SNP/InDel sites, and a total of 31 haplotypes having more than 10 individuals were selected (Fig. 5b). As shown in Fig. 7a, three haplotypes, H_3, 7 and 28, contained 90.78% of the *japonica* individuals, while 93.88% of the *indica* individuals were in the other haplotypes. This finding suggested that SNPs at nt 35, 93, 242, 412, 447, 537, 853, 1164 and 1247 of the *sd1* promoter region (Fig. 5b) clearly differentiated between *japonica* and *indica*. Most accessions in H_21, 24, 29, 23 and 27 are ‘others,’ suggesting that SNPs at nt 35, 734, 759, 820, 1133, 1137 and 1345 differentiated between *indica* and others. Furthermore, no SNP or haplotype was found associated with PH in Fig. 7b, indicating that the PH of rice is mostly controlled by the SD1 protein function but not the gene expression level.

## Discussion

Wild rice is a crucial germplasm resource not only for cultivated rice breeding but also rice domestication studies. PH is a complex trait controlled by multiple genes. Although numerous dwarf mutants in rice have been described during the past decades, the exact functions of genes from wild relatives remains unclear [4, 8]. In this study, we described a novel allele of a classic rice dwarf mutant, *sd1*, which was first described as a “green revolution” gene in the 1960’s [8, 20]. CSSLs are an excellent platform for studying wild alleles in cultivated rice genetic backgrounds. In our laboratory, a set of CSSLs of wild rice was constructed and used for gene discovery, resulting in the discovery of many QTLs related to important agronomic traits [10, 17]. In the current study, a main PH QTL, *qCL1.2,* was identified. *qCL1.2* was detected in four environments. A NIL of *qCL1.2*,CSSL28,had a greater PH than the recurrent parent (Fig. 1), and the F_2_ population demonstrated a perfect 3:1 segregation ratio. These data showed that *qCL1.2* was a dominant locus that can significantly promote PH. Using the genetic segregation populations, the location of *qCL1.2* was narrowed to a 131-kb interval. The “green revolution” gene *sd1* (*LOC_Os01g66100*) was identified in this region. One SNP found in the third exon led to translational termination. We also designed special primers for *LOC_Os01g66100* detection in the CSSL28/9311 F_2_ population. All the individuals harboring the wild rice allele showed greater PH values than individuals harboring the 9311 allele (data not shown). This observation confirmed that *qCL1.2* was the wild allele of the *SD1* gene.

The rice genome carries at least two *GA20ox* genes (*GA20ox-1* and *GA20ox-2*). *SD1* corresponds to *GA20ox-2* and plays essential roles in GA biosynthesis and signal transduction processes [8, 21]. There were many nucleotide changes in the promoter sequences of *qCL1.2* (*SD1*) between the two parents (Fig. S3). Although the transcript levels of *SD1* differed between CSSL28 and 9311, the expression patterns in the two parents were similar (Fig. 4), indicating that the CSSL28 phenotypic changes were mainly caused by changes in the SD1 protein’s function. Three genes involved in GA signaling, *EUI1*, *SLR1* and *GID1*,were expressed at significantly higher levels in 9311 than CSSL28 at 15 d after germination. *GID1* encodes a soluble receptor for GA [20], and SLR1 is a rice DELLA protein that binds to GID1 [22, 23]. Both GID1 and SLR1 undergo negative feedback regulation by GA signaling, as well as*SD1* and*GA20ox* genes. We deduced that the high PH of CSSL28 was induced by active SD1, and feedback regulated by the GID1–SLR1 pathway through GA signaling. *EUI1*, encoding a putative cytochrome P450 monooxygenase, regulates internode elongation by modulating GA responses in rice. Overexpression of *EUI1* gave rise to the GA-deficient-like phenotypes [24]. CSSL28 had elongated internodes compared with 9311(Fig. 1), which might be regulated by active SD1 through repressed*EUI1*. A transgenic experiment should performed to confirm the exactly function of this wild allele.

Plant architecture was an essential target of artificial selection during both rice domestication and is still a target of modern breeding. In this study, two SNPs of *SD1*, nt 299 in the first exon (A/G, E to G) and 1019 in the third exon (A/G, Q to R), clearly differentiated*japonica* and *indica*. These two SNPs were firstreported by Asano et al. (2011) as key natural variations involved in rice domestication [19]. The results here were consistent with those of Asano et al. (2011). However, these two SNPs were not associated with PH. Most *japonica* accessions carried *SD1-EQ* (in the right group of Fig. 6) and had high PH values. Almost all the *indica* accession in the left group of Fig. 6 had short PH values. In the right group of Fig.6, the eight haplotypes of mostly short individuals indicated that eight InDels, 1-bp deletions at nt 301, 302, 303, 318, 387, 456, 564 and 577, had been specifically selected during *indica* domestication (Fig. 5a, red rectangle). In H_13 and 14, most of the samples were *japonica* and carried A at nt 299 and G at nt 1019; this is an intermediate allele *SD1-ER*, which was not identified in Asano et al.’s paper (2011). This discrepancy may be due to that fact that they used only 72 rice accessions, while we used more than 2800 landraces, including 854 *japonica* and 1789 *indica*. For H_20, most of the accessions were short, although it appeared in the middle group of Fig. 6, because the FNP at nt 1026 led to the translational termination of SD1.

In the promoter region, only 3 haplotypes were represented in *japonica*, while there were 28 haplotypes in *indica* rice. This finding was consistent with Asano et al. (2011) who determined that the nucleotide diversity of the *SD1* flanking region in *japonica* was much lower than in *indica*. However, no SNP was associated with PH, indicating that artificial selection only occurred for the *SD1* coding region during the differentiation of *japonica* and *indica*. Additionally, it is the distinct *SD1* alleles, not their expression levels, that played an active role in PH during rice domestication. Our results revealed a new allele of the “green revolution” gene *SD1* from wild rice, which increased the PH in a NIL. Eight InDels and one FNP in the *SD1* coding region were selected during rice domestication, in parallel with *japonica* and *indica* differentiation. Our study provides new insights into the functions and evolution of this gene.

## Conclusions

In this study, a novel allele of SD1 gene was identified from wild rice using a set of CSSLs. The wild allele of *SD1*can significantly promote rice internode length by regulating the expression levels of genes involved in gibberellin biosynthesis and signal transduction. Two key FNPs as key natural variations involved in rice domestication were previously reported, our findings provide new evidence for artificial PH selection during rice domestication and differentiation. The novel *SD1* allele and the new FNPs found in this study provide an increased understanding of rice PH-targeted breeding.

## Methods

### Plant material and field trial

A set of 198 CSSLs produced from common wild rice (*O. rufipogon*) as the donor and an elite *indica* variety, 9311, as the recurrent parent was developed in our laboratory as previously reported [10]. The CSSLs and 9311 were grown under five environmental conditions as shown in Table 1. Each plot consisted of rows having 10 plants. In total, 40 plants of each genotype in each plot were planted with a 10 × 27-cm spacing. Crop management and disease and pest control were carried out in accordance with local recommendations.

### Phenotypic survey and histological observations

The PH was measured from the ground surface to the tallest panicle. Internodes from top to bottom were named P (panicle), first through sixth. The internodes of each stem at the mature stage were fixed in a FAA solution, containing 50% ethanol, 5% acetic glacial and 3.7% formaldehyde, for 24 h at 4 °C and were then dehydrated in a graded ethanol series (70, 80, 90 and 100% twice). The microscopic images were captured by a Leica Digital Camera system. Stem cuticles were prepared for light microscopic observations according to standard preparation techniques [25].

### Gene expression analysis

The expression materials CSSL28 and 9311 were planted in an artificial climate chamber (model: XT5408-CC320TL2H, Xutemp Tech Compay, Hangzhou, China), and the humidity was stably controlled at 80% ± 5%.An 8-h light/12-h dark photocycle was used, and the temperature was controlled at ~ 28 °C.After 5 d of hydroponic culturing in a light incubator, the culturing was continued in a mixed nutrient soil, to ensure uniform growth conditions. Sampling was carried out at 5, 15 and 30 d of culturing. Liquid nitrogen was immediately injected to prevent RNA degradationafter sampling. RNA was extracted using TRIzol reagent (Invitrogen, CA, USA) and treated with DNase I (Invitrogen).cDNA was synthesized using SuperScript III Reverse Transcriptase (Invitrogen). A quantitative analysis of gene expression was performed on an Applied Biosystems 7500 Real-Time PCR System using SYBR Premix Ex Taq (TaKaRa, Otsu, Japan). Data were analyzed using a relative quantitative method [26]. Each real-time PCR reaction had three duplications, and the *Actin* gene of rice was used as an internal reference.

### DNA extraction, PCR protocol and molecular marker analysis

DNA was extracted from rice seedling individuals as previously described [27]. The PCR reaction volume was 15 μL, containing 1.2 μL of template DNA, 0.075 μL of Taq DNA polymerase, 1.5 μL of 10× buffer, 0.3 μL of 10 mM dNTP, 0.6 μL of 10 μmol/L forward and reverse primers and 10.725 μLddH_2_O.After 5 min of pre-denaturation at 95 °C, 33 cycles of 94 °C for 30 s, 56 °C for 30 s and 72 °C for 30 s were performed, followed by 72 °C for 7 min. The PCR products were electrophoresed on a 4% polyacrylamide gels and visualized by silver staining.

The SSR primers used in this study werepreviously published [28, 29], InDel primers were designed in our laboratory [16]. The other primers used in the experiment were based on the 9311 reference genome sequence and were designed online at the NCBI website (Https://www.ncbi.nlm.nih.gov/). Alignments were performed on the Grammer website to ensure the accuracy of the location and the specificity of the primers. Sequences of all molecular markers used in this study are shown in Table S2.

### QTL analysis and candidate gene prediction

The analytic softwareQTL IciMapping [30] was used to processes genotypic and phenotypic data for the CSSL population and the offspring. The method used in this studywas a complete interval–additive model, and the LOD threshold was defined as 2.5. Thus, a LOD value greater than or equal to 2.5 indicated that there is a valid QTL at the site. Naming was performed in accordance with the McCouch method [31].

### Network and genetic diversity analyses

Data on the *SD1* gene sequences of 2822 rice accessions were compiled from the Rice Functional Genomics and Breeding Database (http://www.rmbreeding.cn/snp3k) [32]. This sub-database is a global resource that contains tools, such as a polymorphism information retrieval function, genome browser visualization system, and data export system, for specific genomic regions. All the SNPs located in the promoter and coding regions of the *SD1* gene were extracted based on the genome gff3 annotation. The haplotype analysis was performed using Perl scripts, and only non-synonymous SNPs were considered. Numbers of haplotypes and haplotype diversity levels were determined using DnaSPv5 software (http://www.ub.edu/dnasp) and introduced into the NETWORK 5.0program for haplotype network construction [33].

## Supplementary information

**Additional file 1 Fig. S1.** The distribution of PH of CSSLs under five environments (E1–5).

**Additional file 2 Fig. S2. a**, linkage map of SSR/InDel markers used in CSSLs genotyping, the introgrissive segements of CSSL28 were marked as red; **b**, photos of CSSL28 (right) and 9311(lift) seedlings at 5, 15, 30 days after germination (DAG)

**Additional file 3 Table S1.** Gene prediction analysis in delimitation region of qCL1.2

**Additional file 4 Fig. S3.** Comparison of *SD1* between rice lines 9311 and CSSL28.**a** SNPs found in the *SD1* promoter and coding regions. **b** Sequence alignment of the *SD1* gene

**Additional file 5 Table S2.** Infomations of SSR and InDel markers used in this study.

## Data Availability

The datasets used and/or analysed during the current study available from the corresponding author on reasonable request.

## Abbreviations

PH: Plant height
CSSL: Chromosome segment substitution line
QTL: Quantitative trait locus
SSR: Simple sequence repeats
InDel: Insert/deletion
SNP: Single nucleotide polymorphism
FNP: Functional nucleotide polymorphism
PCR: Polymerase chain reaction
