# Supplementary figures and images for: Identification and genetic analysis of qCL1.2, a novel allele of the “green revolution” gene SD1 from wild rice (Oryza rufipogon) that enhances plant height
Source: BMC Genet. 2020 Jun 11;21:62. doi: 10.1186/s12863-020-00868-w (PMC7291570; doi:10.1186/s12863-020-00868-w)

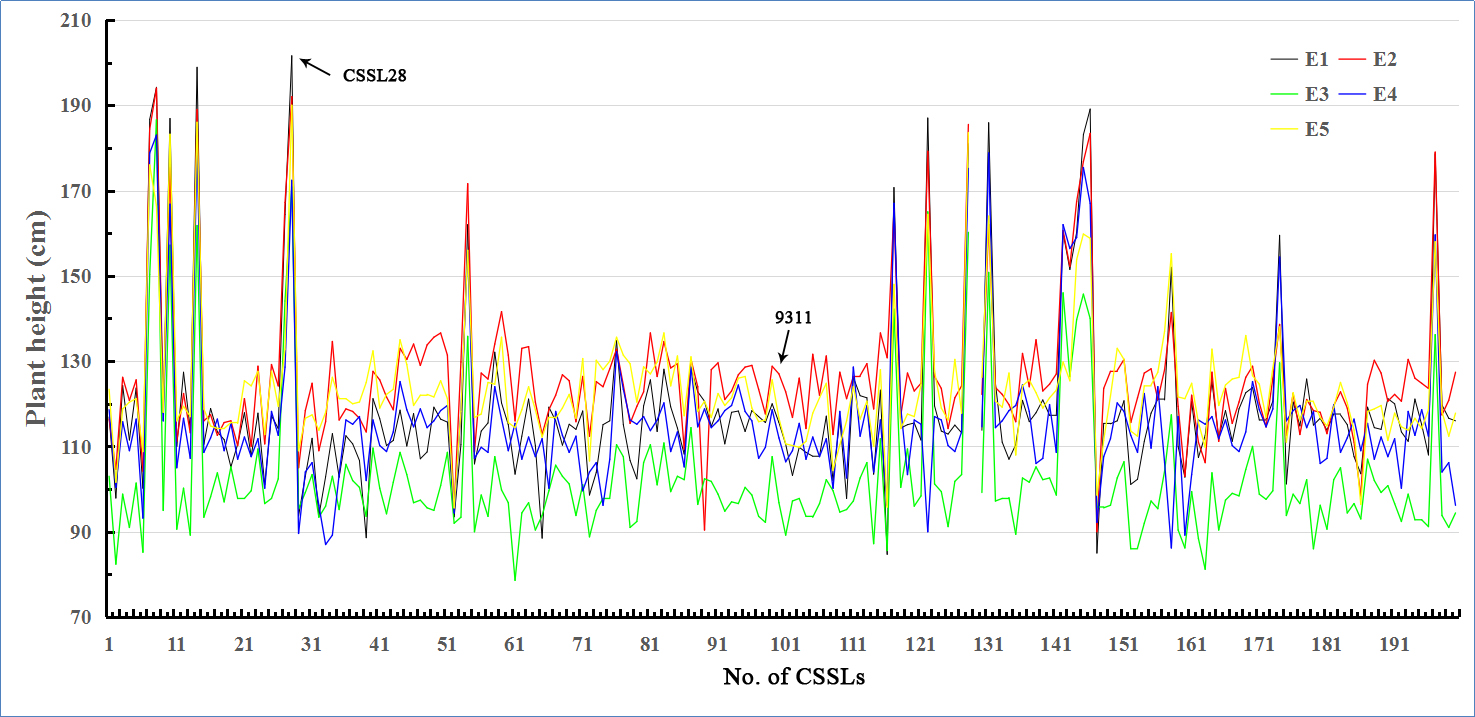

Supplement: Supplementary file 1 — Additional file 1 Fig. S1. The distribution of PH of CSSLs under five environments (E1–5). [file 12863_2020_868_MOESM1_ESM.jpg]

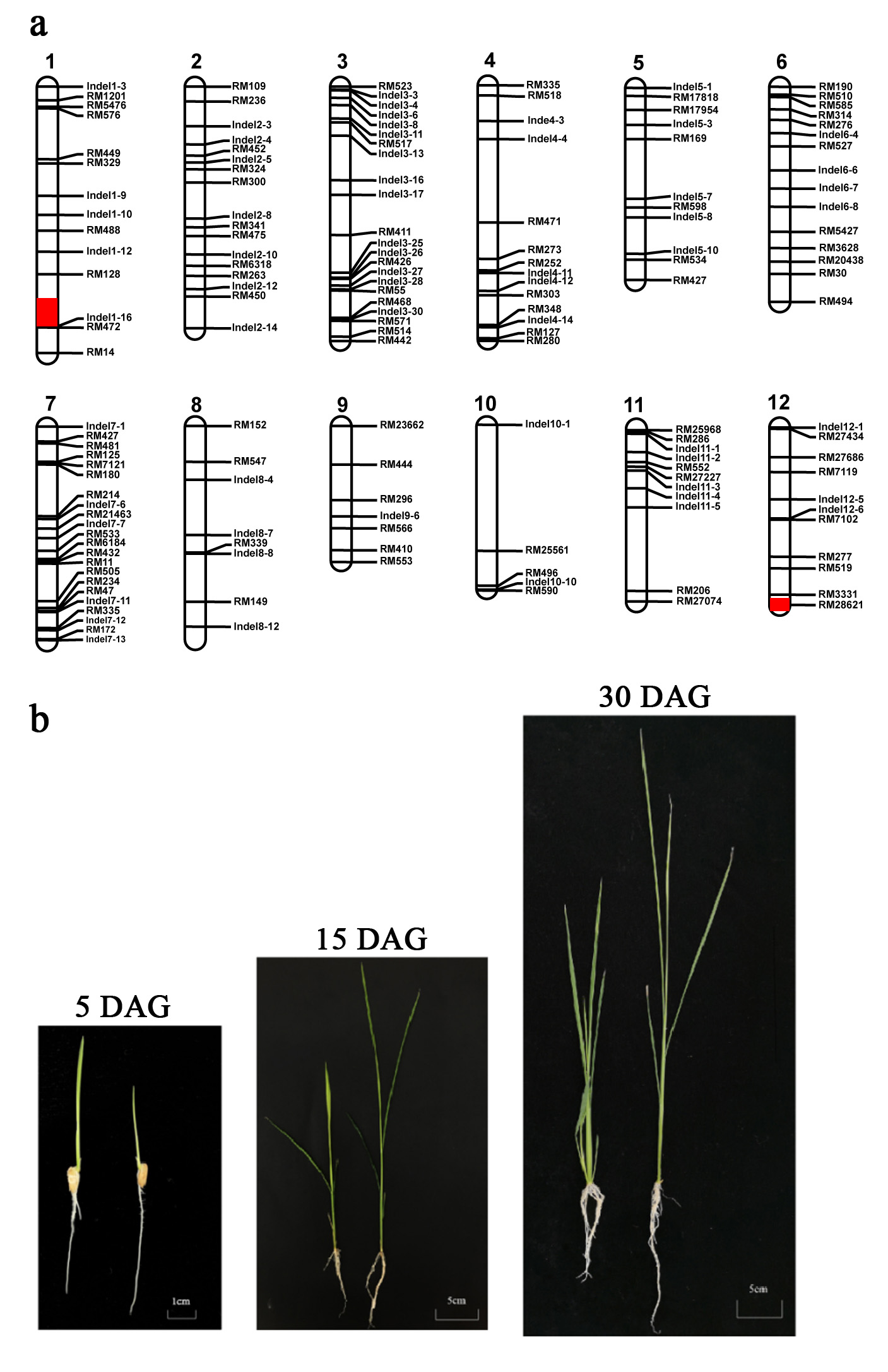

Supplement: Supplementary file 2 — Additional file 2 Fig. S2. a, linkage map of SSR/InDel markers used in CSSLs genotyping, the introgrissive segements of CSSL28 were marked as red; b, photos of CSSL28 (right) and 9311(lift) seedlings at 5, 15, 30 days after germination (DAG) [file 12863_2020_868_MOESM2_ESM.jpg]

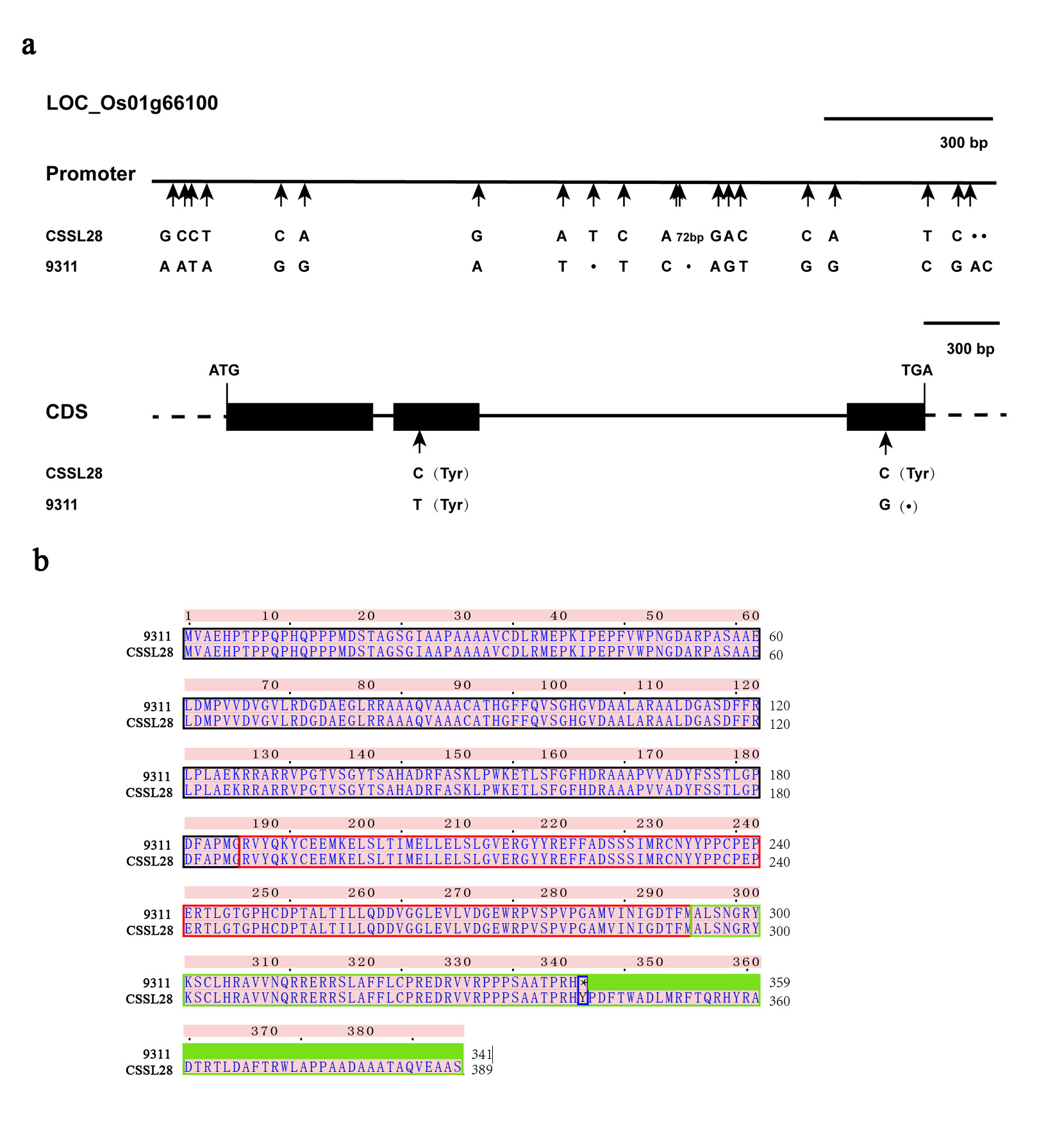

Supplement: Supplementary file 4 — Additional file 4 Fig. S3. Comparison of SD1 between rice lines 9311 and CSSL28.a SNPs found in the SD1 promoter and coding regions. b Sequence alignment of the SD1 gene [file 12863_2020_868_MOESM4_ESM.jpg]
