# Supplementary material for: Identification and genetic analysis of qCL1.2, a novel allele of the “green revolution” gene SD1 from wild rice (Oryza rufipogon) that enhances plant height
Source: BMC Genet. 2020 Jun 11;21:62. doi: 10.1186/s12863-020-00868-w (PMC7291570; doi:10.1186/s12863-020-00868-w)
Supplement: Supplementary file 3 — Additional file 3 Table S1. Gene prediction analysis in delimitation region of qCL1.2 [file 12863_2020_868_MOESM3_ESM.doc]

| RAPDB Locus | MSU Locus | Location(bp) | Annotation | gene symbol |
| --- | --- | --- | --- | --- |
| Os01g0883200 | LOC_Os01g66050 | 38329179 - 38332595 | wound-responsive family protein |  |
|  | LOC_Os01g66060 | 38337612 - 38336217 | expressed protein |  |
| Os01g0883400 | LOC_Os01g66070 | 38344410 - 38344937 | PHD-finger domain containing protein |  |
|  | LOC_Os01g66080 | 38346711 - 38349796 | retrotransposon protein |  |
|  | LOC_Os01g66090 | 38358147 - 38352142 | retrotransposon protein |  |
| Os01g0883800 | LOC_Os01g66100 | 38382382 - 38385504 | gibberellin 20 oxidase 2 | *sd1; OsGA20ox2; qSD1-2* |
|  | LOC_Os01g66110 | 38391780 - 38386267 | methyltransferase |  |
| Os01g0884300 | LOC_Os01g66120 | 38401533 - 38398517 | No apical meristem protein | *OsNAC6; SNAC2* |
| Os01g0884400 | LOC_Os01g66130 | 38409201 - 38413065 | armadillo/beta-catenin repeat family protein |  |
| Os01g0884500 | LOC_Os01g66140 | 38424621 - 38413166 | plus-3 domain containing protein |  |
| Os01g0884700 | LOC_Os01g66150 | 38433670 - 38431286 | expressed protein |  |
| Os01g0884800 | LOC_Os01g66160 | 38435460 - 38437074 | pentatricopeptide |  |
|  | LOC_Os01g66160 | 38440460 - 38436842 | SNARE associated Golgi protein |  |
| Os01g0884850 | LOC_Os01g66170 | 38440460 - 38436842 | SNARE associated Golgi protein |  |
| Os01g0885000 | LOC_Os01g66180 | 38443627 - 38446431 | cytochrome c |  |
| Os01g0885200 | LOC_Os01g66190 | 38450486 - 38447688 | expressed protein |  |
| Os01g0885300 | LOC_Os01g66200 | 38460564 - 38454786 | expressed protein |  |
|  | LOC_Os01g66210 | 38465598 - 38464169 | retrotransposon protein | |

**Table S1 Gene prediction analysis in delimitation region of *qCL1.2***
