# Supplementary material for: Identification and genetic analysis of qCL1.2, a novel allele of the “green revolution” gene SD1 from wild rice (Oryza rufipogon) that enhances plant height
Source: BMC Genet. 2020 Jun 11;21:62. doi: 10.1186/s12863-020-00868-w (PMC7291570; doi:10.1186/s12863-020-00868-w)
Supplement: Supplementary file 5 — Additional file 5 Table S2. Infomations of SSR and InDel markers used in this study. [file 12863_2020_868_MOESM5_ESM.docx]

**Table S2 SSR and InDel markers used in this study**

**Markers used in CSSLs genotyping.**

| **Locus name** | **Chr.** | **Genetic distance(cM)** | **Physical location** | **Forward primer** | **Reverse primer** |
| --- | --- | --- | --- | --- | --- |
| Indel1-3 | 1 | 19.64 | 5302378-5302397 | gggaaatttgggaggaagac | cgagcaagctacccgaatta |
| RM1201 | 1 | 26.51 ­­ | 7158556-7158575 | gctacgtacgagccctagttaccg | taccgcgccacatatacacaacc |
| RM5496 | 1 | 29.91 | 8075306-8075327 | gtagccatagcctttcccaaagc | tctcctgcagctgatttcttgg |
| RM576 | 1 | 30.15 | 8139864-8139885 | gcagtaatatgtggaggtttcg | gtaggtcaaagccttctatcagc |
| RM449 | 1 | 56.01 | 15123035-15123615 | ccttctcccttaattaacccttcc | gattcatcacgagagcacaaagg |
| RM329 | 1 | 57.99 | 15657772-15657791 | cattcggctgctgctattc | gcttgtcacatcttgcacag |
| Indel1-9 | 1 | 74.28 | 20055808-20055827 | tcccctctccacttgtcaac | tttttagtggcatagatcctcttc |
| Indel1-10 | 1 | 83.79 | 22624360-22624379 | gttcaggcaattccatcgtt | ctaattcgcgaaacgaatct |
| RM488 | 1 | 91.88 | 24808552-24808571 | aacaaccagcgtatgcgttctcg | cccacggctttgtaggaagaagc |
| Indel1-12 | 1 | 102.34 | 27632744-27632763 | ccacaagctcaagcttcaaa | tcgaattactcgcattgatcc |
| RM128 | 1 | 113.85 | 30738749-30738773 | tgatttcttggaagcgaagagtgagg | cctccttgtgctcagccatgc |
| Indel1-16 | 1 | 139.66 | 37707816-37707835 | ttcatatccgcaggcaattt | gcctttctgttcatggcagt |
| RM472 | 1 | 144.56 | 39030807-39030826 | ttaggcgatcgatctcatctctcc | cagcttcccgtgagtagcaacg |
| RM14 | 1 | 153.2 | 41363934-41363953 | cattgacgtggcactttgttcc | agagagcacgcaatggagtatgc |
| RM109 | 2 | 0.68 | 183311-183332 | agccaagatcgtcttcatctctcg | tcgtctccttcttccttcttcttcc |
| RM236 | 2 | 7.8 | 2105603-2105623 | gtgaagcacatgtggctagttgc | ttccctcaagaatctgtgtcttcc |
| Indel2-3 | 2 | 20.29 | 5477492-5477511 | atagggtgggtgtgctgaac | gcacaaaactgcaggtctcc |
| Indel2-4 | 2 | 29.58 | 7985272-7985291 | agtgtcccaagcgagaaaac | atgcacgagtgagtgtgagc |
| RM452 | 2 | 35.42 | 9563307-9563327 | gtggacttggcgagatgctacg | gttaagggcagccaccagatcg |
| Indel2-5 | 2 | 38.75 | 10461142-10461161 | gggaacttctctcccacaca | cacgtacacgttttccgaac |
| RM324 | 2 | 42.18 | 11389704-11389942 | gattccacgtcaggatcttctgg | gctcaccagttgagattgaaagg |
| RM300 | 2 | 48.86 | 13191386-13191406 | gggcttaaggacttctgcgaacc | agcgatccacatcatcaaatcg |
| Indel2-8 | 2 | 66.99 | 18087595-18087614 | ccatcagcatcagcaatagg | gaatatgttgtggagaccaatatatga |
| RM341 | 2 | 71.64 | 19342016-19342033 | caagaaacctcaatccgagc | ctcctcccgatcccaatc |
| RM475 | 2 | 75.57 | 20405168-20405187 | cctcacgattttcctccaac | acggtgggattagactgtgc |
| Indel2-10 | 2 | 85.37 | 23050849-23050866 | ggctggctgttgctcatc | aaaaatcccaaccctgctg |
| RM6318 | 2 | 90.47 | 24426463-24426482 | aagtgcctcgaattacacatctcc | gctgcttctgtccagtgagacc |
| RM263 | 2 | 95.82 | 25871271-25871290 | aatctatggacctgggaggaacc | tgacgagagtgctacgtttgagc |
| Indel2-12 | 2 | 102.5 | 28078470-28078489 | aagaaagagaatgccgcaca | gcgactagctctcagccatc |
| RM450 | 2 | 106.05 | 28634075-28634094 | cagtagtacgccggatcaacagg | ccacttgttccatccacatctcc |
| Indel2-14 | 2 | 122.49 | 33072462-33072481 | agtgaaatttgagcccaacg | taaaagcaaaggccgaaaaa |
| RM523 | 3 | 4.89 | 1320598-1320617 | tgaattcttgcacatggtcagc | tgggaggtttgctagggtaatcc |
| Indel3-3 | 3 | 6.42 | 1732991-1733012 | tggtttatattggaacggagga | gttacatgccctttcgcagt |
| Indel3-4 | 3 | 6.43 | 1735365-1735385 | gcttaccacacctctcctcct | tccatatgcttccttcttcca |
| Indel3-6 | 3 | 10.69 | 2885993-2886010 | gtttacgaatgaaccagt | ctcattgaggcaaaggac |
| Indel3-8 | 3 | 14.41 | 3889740-3889759 | taatttcggctcatccaagc | gaagctccgcaggttcag |
| Indel3-11 | 3 | 20.94 | 5654369-5654388 | ggaatccctcccttcttgtc | ggtcggtaaagacggtgaaa |
| RM517 | 3 | 22.84 | 6167007-6167026 | cagctccttcctatccgtctcc | tcagatctagccgagaaatcaagg |
| Indel3-13 | 3 | 29.64 | 8003333-8003352 | gccattgatcttctgcaggt | tttgttgtcaatgccctgtt |
| Indel3-16 | 3 | 52 | 14041082-14041101 | cgacgctgttgatcctgtta | gaaattaagcagcggaagca |
| Indel3-17 | 3 | 59.43 | 16047152-16047171 | gcatccatggttgagattcc | tgcgctgctaaatgaaaaga |
| RM411 | 3 | 79.37 | 21430734-21430754 | gtaggaaattcttcgccagatgc | ccgagacttggaacaatcttaggc |
| Indel3-25 | 3 | 99.56 | 26880984-26881003 | gttaggcctgcacttttgga | gacatcaatcttggggagga |
| Indel3-26 | 3 | 101.38 | 27373442-27373461 | gtgatggtgaggggatctca | atccctcctcccttgctg |
| RM426 | 3 | 102.21 | 27595689-27595709 | catcgccgaaatccatcttcc | aaggcccatttcattgtagagtgc |
| Indel3-27 | 3 | 105.08 | 28371057-28371076 | tgggctattattgggctttg | cgtgggataaaaccaccaag |
| Indel3-28 | 3 | 107.22 | 28948637-28948656 | tgcgcgtgaaaaataagaaa | ccatgcttagccgctacact |
| RM55 | 3 | 107.6 | 29052279-29052297 | ccgtcgccgtagtagagaag | tcccggttattttaaggcg |
| RM468 | 3 | 121.04 | 32681981-32682000 | aaagatccgtgtcctcaatcagc | cctaaagcccttccttgttgtgg |
| Indel3-30 | 3 | 122.26 | 33010205-33010224 | gccatcttgccatttgttct | ctctgcttttgctgcctctt |
| RM571 | 3 | 122.81 | 33157687-33157713 | ggaggtgaaagcgaatcatg | cctgctgctctttcatcagc |
| RM514 | 3 | 130.7 | 35288369-35288389 | cttctcagattgatctcccattcc | gggagagaggaagaagacaagg |
| RM442 | 3 | 132.55 | 35788693-35788714 | cttaagccgatgcatgaagg | atcctatcgacgaatgcacc |
| RM335 | 4 | 2.55 | 688353-688466 | gtacacacccacatcgagaagc | tccatggatatacgaggagatgc |
| RM518 | 4 | 7.54 | 2034737-2034763 | aagacacaagcaaacagctcaacc | aagcttgcttggttcaagagagg |
| Indel4-3 | 4 | 20.85 | 5630637-5630656 | ggtcgctggttcttgaatgt | aatggtttgacttcgaccaaa |
| Indel4-4 | 4 | 30.53 | 8242691-8242711 | ctcacagtttctaggcggaaa | agccgagtagggctgaataa |
| RM471 | 4 | 70.36 | 18996786-18996805 | agaaatggatcggactgaacatgc | agacactcggacgcacaagc |
| RM273 | 4 | 89.07 | 24048269-24048289 | gaagccgtcgtgaagttacc | gtttcctacctgatcgcgac |
| RM252 | 4 | 93.94 | 25364256-25364277 | ttcgctgacgtgataggttg | atgacttgatcccgagaacg |
| Indel4-11 | 4 | 95.14 | 25687553-25687572 | tgaacacatcccaattgctt | tagacgagaggggttggaga |
| Indel4-12 | 4 | 104.4 | 28188053-28188072 | cgtggcaatatggttccttt | tcggatacgtaaaacggaaaa |
| RM303 | 4 | 106.52 | 28760131-28760151 | gcatggccaaatattaaagg | ggttggaaatagaagttcggt |
| RM348 | 4 | 121.74 | 32869438-32869458 | catgaagctgtgttgctgttgc | cgctactaatagcagagagaccatcg |
| Indel4-14 | 4 | 122.74 | 33140151-33140170 | ggatggtgaggtgaggtgtt | cgtgttttctcccccaatc |
| RM127 | 4 | 128.57 | 34714843-34714864 | cgaagctttcggtgggatagc | accttgagcgagtccttgaacg |
| RM280 | 4 | 130.28 | 35174676-35174694 | gtgctctccatgtcggattatgc | caaggcaacaagattggttagtgg |
| Indel5-1 | 5 | 2 | 541032-541051 | ccttgatcgattgctctggt | actttctccgtgttgcttgc |
| RM17818 | 5 | 5.98 | 1613753-1613774 | ttgcctcatgtttgcttcatcc | agctgacaacgacgacactgc |
| RM17954 | 5 | 13.52 | 3651562-3651585 | atttcagtacaaggcacccatgc | gtagacgagggagtaccaacttgc |
| Indel5-3 | 5 | 20.54 | 5546655-5546674 | gctcccctcaacttttcctc | tcggttgcctgaataccttt |
| RM169 | 5 | 27.77 | 7497975-7497997 | cacctcctccaagatccttatgc | ctctctgtctcgctgtctgttgc |
| Indel5-7 | 5 | 58.35 | 15754411-15754430 | aaatttaggccaggcagctt | tctctcacacgcttattcatcttt |
| RM598 | 5 | 62.27 | 16811550-16811570 | tttccggacagctggattatagc | gattgaggcagagagacctaattcc |
| Indel5-8 | 5 | 67.59 | 18248303-18248321 | cgtgccgatgacaaacttc | gaggatccatgtccaccatt |
| Indel5-10 | 5 | 85.99 | 23218061-23218080 | tcgcattgagatttgtgcat | tcgtaaccacactgcaactg |
| RM534 | 5 | 89.15 | 24070248-24070267 | ttcgaactggagcttcttcttgg | aacgcaactgacacagactgacc |
| RM274 | 5 | 99.67 | 26910796-26910816 | cctcgcttatgagagcttcg | cttctccatcactcccatgg |
| RM190 | 6 | 6.54 | 1765739-1765761 | ctttgtctatctcaagacac | ttgcagatgttcttcctgatg |
| RM510 | 6 | 10.49 | 2832512-2832531 | gtttgacgcgataaaccgacagc | atgaggacgacgagcagattcc |
| RM585 | 6 | 11.74 | 3169525-3169546 | cagtcttgctccgtttgttg | ctgtgactgacttggtcatagg |
| RM314 | 6 | 17.56 | 4740332-4740346 | ctagcaggaactcctttcagg | aacattccacacacacacgc |
| RM276 | 6 | 23.07 | 6230045-6230185 | gtcctccatcgagcagtatcagc | ctagcaagacatggacctcaacg |
| Indel6-4 | 6 | 29.73 | 8027637-8027658 | cagttaacaccaatccaatcca | ccaaatgggcagtagtttgaa |
| RM527 | 6 | 36.53 | 9862291-9862523 | cggtttgtacgtaagtagcatcagg | tccaatgccaacagctatactcg |
| Indel6-6 | 6 | 48.77 | 13168366-13168385 | cctcatccaggggtcatgta | cggtcaagtgtcatccaggt |
| Indel6-7 | 6 | 57.85 | 15618560-15618579 | gcgattattgagagcgagga | gcctcttgtgggaagaacaa |
| Indel6-8 | 6 | 67.29 | 18168579-18168598 | tcacctttatggtgccgaag | gaagctgcttttgcttccac |
| RM5427 | 6 | 79.7 | 21519085-21519226 | tgctgttgacacttgacaggtagc | cacaattattgcggctcatcg |
| RM3628 | 6 | 87.92 | 23737032-23737157 | gccctagacacacccgtacc | tgccagatcagaaatcatgc |
| RM20438 | 6 | 94.71 | 25571372-25571394 | cacaacgaatgtggtgtgtcc | cgacatagatgaggccctattcc |
| RM30 | 6 | 100.94 | 27253359-27253378 | ggttaggcatcgtcacgg | tcacctcaccacacgacacg |
| RM494 | 6 | 115.15 | 31089144-31089163 | gggatcgagatagacatagacc | tctgtacagtgtcattccttcc |
| Indel7-1 | 7 | 2.42 | 654253-654274 | tgactgttacccttacgtgcag | cgggatgaaacagattctgag |
| RM427 | 7 | 9.92 | 2679665-2679685 | ttgagctgatgagagttggttgc | ctgtcactagctctgccctgacc |
| RM481 | 7 | 10.65 | 2876314-2876333 | tagctagccgattgaatggc | ctccacctcctatgttgttg |
| RM125 | 7 | 20.3 | 5480476-5480497 | atcagcagccatggcagcgacc | aggggatcatgtgccgaaggcc |
| RM7121 | 7 | 20.83 | 5622808-5623030 | taccagctgcatgttacccgatacc | cggaatcaaattccagcaacagc |
| RM180 | 7 | 21.25 | 5736273-5736303 | ccttctccttctttcagcttctgc | caacttgctctacttgtggtgagg |
| RM214 | 7 | 47.35 | 12784610-12784633 | ctgatgatagaaacctcttctc | aagaacagctgacttcacaa |
| Indel7-6 | 7 | 49.32 | 13315866-13315884 | ccccatgaggcctacactt | agcagcataatcagatgagacg |
| RM21463 | 7 | 53.72 | 14505672-14505694 | gttgatctggagtcgatgagtgc | cccacgaaagatgctttaagacg |
| Indel7-7 | 7 | 58.76 | 15864592-15864610 | atcggtgccgctcctagat | cactccacagacatgcaattt |
| RM533 | 7 | 64.86 | 17512976-17512996 | aaaggccgtacctttgccttcc | agctagggatccatcctccaacc |
| RM6184 | 7 | 69.22 | 18689140-18689163 | agtcgtacaggctgccgttctcc | acatgctcctccacgacaagagc |
| RM432 | 7 | 70.22 | 18959592-18959611 | ctttctgtctcacgctggattgg | tgcgtacgtgatgaatggttagc |
| RM11 | 7 | 71.32 | 19256914-19257039 | atcggtgcttggctggatagc | ccaccttcttctcctcctcttcc |
| RM505 | 7 | 90.84 | 24527750-24527771 | agagttatgagccgggtgtg | gatttggcgatcttagcagc |
| RM234 | 7 | 94.35 | 25473795-25473814 | ttcagccaagaacagaacagtgg | cttctcttcatcctcctccttgg |
| RM47 | 7 | 95.58 | 25807730-25807748 | actccactccactccccac | gtcagcaggtcggacgtc |
| Indel7-11 | 7 | 96.04 | 25931538-25931558 | gcccacctgtcattgagagta | gtttttgcgcttttgttgct |
| RM1335 | 7 | 104.81 | 28299825-28299844 | gcgccattcttgtcatctaattgc | atcgaacaagaagagtggcttgg |
| Indel7-12 | 7 | 105.44 | 28469598-28469617 | cgttcgtgtttttcgctgat | gatcggaggcttttgtttga |
| RM172 | 7 | 109.49 | 29562421-29562443 | tgcagctgcgccacagccatag | caaccacgacaccgccgtgttg |
| Indel7-13 | 7 | 109.86 | 29663271-29663289 | tggaggctttctcgctttc | gaggaagtcgaggatgagga |
| RM152 | 8 | 2.53 | 683962-683983 | gaaaccaccacacctcaccg | ccgtagaccttcttgaagtag |
| RM547 | 8 | 20.71 | 5592402-5592422 | ttgtcaagatcatcctcgtagc | gtcattctgcaacctgagatcc |
| Indel8-4 | 8 | 29.85 | 8060517-8060539 | cgtgcggcttacaagagata | tgaggcactaatcatcttctctg |
| Indel8-7 | 8 | 57.77 | 15598198-15598216 | tttttaccgtgtcggtactgc | ctccaaaacacgggaccat |
| RM339 | 8 | 66.46 | 17945181-17945202 | gtaatcgatgctgtgggaag | gagtcatgtgatagccgatatg |
| Indel8-8 | 8 | 67 | 18089671-18089693 | ccctccatgttgtgagttcc | tgattagatccagaaagggagaa |
| RM149 | 8 | 91.57 | 24724078-24724103 | gctgaccaacgaacctaggccg | gttggaagcctttcctcgtaacacg |
| Indel8-12 | 8 | 104.15 | 28119245-28119264 | tgaattgaacctccgtcctc | agaactgcaccacgaagctc |
| RM23662 | 9 | 1.6 | 431978-431999 | gagaggacgatggcactattgg | cgaggaacttgattcgcatgg |
| RM444 | 9 | 21.95 | 5926291-5926310 | tgcatctttcaccgtagtcctagc | cttgctggagctcgtagatgc |
| RM296 | 9 | 39.94 | 10785115-10785132 | cacatggcaccaacctcc | gccaagtcattcactactctgg |
| Indel9-6 | 9 | 48.34 | 13052791-13052810 | gcatgtatcgtggacatgga | tccttgatcaacaccgtcaa |
| RM566 | 9 | 54.47 | 14705799-14705818 | aatatggtggcgcgtacatcc | tgatcgagccaacaacaactgg |
| RM410 | 9 | 65.35 | 17644023-17644043 | gctagattcacgggccttgc | gtgcgttcggatggaggtagg |
| RM553 | 9 | 71.57 | 19325086-19325105 | tgtgtggccactttactcaacc | ggagaaggtggttgcagaagc |
| Indel10-1 | 10 | 1.95 | 525770-525790 | aattcttatggacggatacgc | tcagcatctcgtaagcaaaaa |
| RM25561 | 10 | 65.65 | 17726481-17726503 | tactaccttccacgcttccatcc | gcgatttcttcgggagttaggg |
| RM496 | 10 | 83.34 | 22501660-22501687 | gacatgcgaacaacgacatcc | ctatagttgttgcacatgcgatcc |
| Indel10-10 | 10 | 85.57 | 23105161-23105180 | catgctacagagagggaagc | acggcgtctttcggtgtc |
| RM590 | 10 | 85.61 | 23114684-23114702 | catctccgctctccatgc | ggagttggggtcttgttcg |
| RM25968 | 11 | 1.3 | 351918-351941 | cagatagctggtagtcattgtcc | caacttcctataccagtcaacacc |
| RM286 | 11 | 1.43 | 384938-384958 | ctggcctctagctacaaccttgc | aaactctcgctggattcgatagg |
| Indel11-1 | 11 | 3.05 | 822379-822400 | acaaagtctaaggcctgaaaaa | gatgtcttccgggtgagcta |
| Indel11-2 | 11 | 12.41 | 3349998-3350020 | tgatgagctctcacttgttgaaa | cgtacattggcttatgtgatctg |
| RM552 | 11 | 17.94 | 4843013-4843207 | cgcagttgtggatttcagtgc | tcatgctcaacgtttgactgtcc |
| RM26227 | 11 | 19.8 | 5345786-5345808 | caccggagaccagagtagaacg | ctcaaactgatcgaggctgacc |
| Indel11-3 | 11 | 21.71 | 5861035-5861054 | gtgaattcatgacgcgaaga | agcttgatggatgctcaggt |
| Indel11-4 | 11 | 31.04 | 8380755-8380774 | tgagatgtggccattaagga | tggcaaaagatcttatatttacttcg |
| Indel11-5 | 11 | 40.5 | 10934775-10934794 | tctcctcaaaatgggacacc | ataacgcgggacacagaatc |
| RM206 | 11 | 83.26 | 22480807-22480827 | atcgatccgtatgggttctagc | gtccatgtagccaatcttatgtgg |
| RM27074 | 11 | 88.82 | 23980684-23980707 | ggaggagatatcagcaataagagg | gaagaagaatcgtggataggc |
| Indel12-1 | 12 | 2.05 | 552745-552766 | tctggagagctgcagaaaca | gcaatctctgcactttgatacc |
| RM27434 | 12 | 2.58 | 695703-695725 | gtgctgatccacctgacttctcc | ttgggctcggtttctctcttagc |
| RM27686 | 12 | 17.38 | 4693713-4693735 | atgggaacaaccttatcgtctgc | gagagttgggcttcttgttgagg |
| RM7119 | 12 | 24.8 | 6695668-6695687 | ctgagaccatgacgggataaacacc | ggcctcagatcatcacaacttgg |
| Indel12-5 | 12 | 38.8 | 10475651-10475670 | tgggcaactgaatctaacca | ggagatgatgatgcggtgat |
| Indel12-6 | 12 | 48.33 | 13050153-13050176 | caactaaaaccaacacaaaatcca | tgtctagttgcatgtctgagtgtc |
| RM7102 | 12 | 48.93 | 13211325-13211536 | gggcgttcggtttacttggttactcg | ggcggcataggagtgtttagagtgc |
| RM277 | 12 | 67.85 | 18319022-18319039 | cggtcaaatcatcacctgac | caaggcttgcaagggaag |
| RM519 | 12 | 73.72 | 19903791-19903912 | aatttccgcgaaatcagcatcc | tcatctggacagtcgaggtacgc |
| RM3331 | 12 | 87.02 | 23494367-23494386 | acgagagggaggagagagaaacg | ggagagccacaggaacagatcg |
| RM28621 | 12 | 92.5 | 24975839-24975860 | gtgtcaactgtcaacaaacacc | aaggtcagggttacatgatagg |

**Markers used in fine mapping**

| **Locus name** | **Chr.** | **Genetic distance(cM)** | **Physical location** | **Forward primer** | **Reverse primer** |
| --- | --- | --- | --- | --- | --- |
| RM128 | 1 | 113.82 | 30738749-30738773 | tgatttcttggaagcgaagagtgagg | cctccttgtgctcagccatgc |
| InDel1-14 | 1 | 121 | 32677239- | acgctgaaaagcaaggatgt | ttctagccctcctctttgaca |
| RM486 | 1 | 129.44 | 34949898-34949925 | gcttgcattatgcgattgtactcc | tgagctttctcaacaacgactgc |
| RM5389 | 1 | 132.32 | 35726691-35726718 | tgtgctattgcgcgagattatcc | catcaccgctccaactcatgc |
| RM11908 | 1 | 137.7 | 37179868-37179903 | agcggcaacagtgtcgtctcg | gccaatgaggcagcaacagc |
| RM11928 | 1 | 139.17 | 37577314-37577373 | taaaccagatcatgccctcatcc | agcagtaacggttgggtacttgg |
| InDel1-16 | 1 | 139.66 | 37707816-37707835 | ttcatatccgcaggcaattt | gcctttctgttcatggcagt |
| RM11960 | 1 | 141.03 | 38079326-38079385 | gtaattacccatgcacccatgc | catcagttatcatgggcacactacc |
| RM11974 | 1 | 141.99 | 38337085-38337106 | tacgaagtgccaaccactcatgc | caagagattaaatgtccggcttgg |
| RM11982 | 1 | 142.48 | 38468416-38468439 | cgcgttctcccttctttctactcg | gttacatgggcctccagcatagg |
| RM11986 | 1 | 142.95 | 38597143-38597164 | tcaacctcggtgttcctctaagc | agcgacacctcaactctgacagg |
| RM472 | 1 | 144.56 | 39030807-39030826 | ttaggcgatcgatctcatctctcc | cagcttcccgtgagtagcaacg |
